# Metformin inhibits estrogen‐dependent endometrial cancer cell growth by activating the AMPK–FOXO1 signal pathway

**DOI:** 10.1111/cas.13083

**Published:** 2016-11-25

**Authors:** Jingfang Zou, Liangli Hong, Chaohuan Luo, Zhi Li, Yuzhang Zhu, Tianliang Huang, Yongneng Zhang, Huier Yuan, Yaqiu Hu, Tengfei Wen, Wanling Zhuang, Bozhi Cai, Xin Zhang, Jiexiong Huang, Jidong Cheng

**Affiliations:** ^1^ Departments of Internal MedicineThe First Affiliated Hospital of Shantou University Medical CollegeShantouChina; ^2^ Departments of PathologyThe First Affiliated Hospital of Shantou University Medical CollegeShantouChina; ^3^ The Laboratory of Molecular CardiologyThe First Affiliated Hospital of Shantou University Medical CollegeShantouChina

**Keywords:** AMPK, estrogen‐dependent endometrial cancer, FOXO1, metformin, proliferation

## Abstract

Metformin is an oral biguanide commonly used for treating type II diabetes and has recently been reported to possess antiproliferative properties that can be exploited for the prevention and treatment of a variety of cancers. The mechanisms underlying this effect have not been fully elucidated. Our study shows a marked loss of AMP‐activated protein kinase (AMPK) phosphorylation and nuclear human Forkhead box O1 (FOXO1) protein in estrogen‐dependent endometrial cancer (EC) tumors compared to normal control endometrium. Metformin treatment suppressed EC cell growth in a time‐dependent manner *in vitro*; this effect was cancelled by cotreatment with an AMPK inhibitor, compound C. Metformin decreased FOXO1 phosphorylation and increased FOXO1 nuclear localization in Ishikawa and HEC‐1B cells, with non‐significant increase in FOXO1 mRNA expression. Moreover, compound C blocked the metformin‐induced changes of FOXO1 and its phosphorylation protein, suggesting that metformin upregulated FOXO1 activity by AMPK activation. Similar results were obtained after treatment with insulin. In addition, transfection with siRNA for FOXO1 cancelled metformin‐inhibited cell growth, indicating that FOXO1 mediated metformin to inhibit EC cell proliferation. A xenograft mouse model further revealed that metformin suppressed HEC‐1B tumor growth, accompanied by downregulated ki‐67 and upregulated AMPK phosphorylation and nuclear FOXO1 protein. Taken together, these data provide a novel mechanism of antineoplastic effect for metformin through the regulation of FOXO1, and suggest that the AMPK–FOXO1 pathway may be a therapeutic target to the development of new antineoplastic drugs.

**Abbreviations**
Akt: protein kinase B
AMPK: AMP‐activated protein kinase
CAH: complex hyperplasia with atypia (also complex atypical hyperplasia)
CH: complex hyperplasia
compound C: 6‐[4‐(2‐piperidin‐1‐yl‐ethoxy)‐phenyl)]‐3‐pyridin‐4‐yl‐ pyrazolo [1,5‐a]‐pyrimidine
EC: estrogen‐dependent endometrial cancer
FOXO1: human Forkhead box O
IHC: immunohistochemistry
Lkb1: liver kinase b1
NP: normal proliferative endometrium
p‐: phosphorylated
SH: simple hyperplasia

According to the American Cancer Society, endometrial cancer is the most common gynecologic malignancy in women, with 54 870 estimated new cases in the USA in 2015.^1^ Estrogen‐dependent endometrial cancer, also known as type I endometrial cancer, accounts for approximately 80% of cases, is classically of endometrioid histology, and is associated with obesity in up to 90% of cases. Patients with endometrial hyperplasia, or increased glandular proliferation, are at increased risk of developing endometrial cancer.^2^ Hyperplasia include the following three histologic categories: (i) SH; (ii) CH; and (iii) CAH.^3^ Known risk factors for this disease include obesity, diabetes mellitus, hypertension and insulin resistance.^4^ Additionally, type II diabetes is associated with an increased risk for the development of EC.^5^, ^6^

The oral biguanide, metformin, is one of the most commonly used hypoglycemic agents in the management of type II diabetes. Epidemiological studies have reported that diabetic patients being treated with metformin have a reduced cancer incidence or improved response to chemotherapy when compared to patients receiving other oral hypoglycemic agents or insulin.^7^, ^8^, ^9^ Preclinical studies have reported a growth static effect of metformin on breast, prostate, ovarian, and EC cell lines, effected both through alterations in glucose metabolism and inhibition of the phosphatidylinositol‐3‐kinase/protein kinase B (Akt)/mammalian target of rapamycin signaling pathway.^10^, ^11^, ^12^, ^13^ Metformin accumulates in the tumor tissue and activates AMPK, which is a central energy sensor and regulator of cellular and whole‐body energy homeostasis.^14^

A recent study reported that AMPK directly phosphorylates FOXO1 and increases FOXO1‐dependent transcription of manganese superoxide dismutase and catalase.^15^ The FOXO1 transcription factor, which belongs to the Forkhead transcription factors of the FOXO subfamily (FOXO1, FOXO3, FOXO4, and FOXO6 in mammals),^16^ is reported to be involved in various cellular functions including proliferation, differentiation, cell survival, glucose metabolism, longevity, and oxidative stress resistance.^17^ The potent activities of FOXO1 are tightly controlled by multiple mechanisms, which include post‐translational modification such as phosphorylation, acetylation, methylation, and ubiquination, subcellular localization, and direct protein–protein interaction and phosphorylation is central to the regulation of mammalian FOXO1 functions. In the phosphorylated state, FOXO1 is excluded from the nucleus and degraded in the cytoplasm in a ubiquitin‐dependent manner. FOXO1 is negatively regulated by Akt, such that dephosphorylated FOXO1 localizes to the nucleus and shows transcriptional activity, whereas Akt‐dependent phosphorylation of FOXO1 protein causes its nuclear exportation and proteasomal degradation.^18^, ^19^ Mutagenesis studies showed that this AMPK‐mediated phosphorylation of FOXO1 is critical for FOXO1 stability and nuclear localization, establishing the molecular basis for the induction of FOXO1 transcriptional activity. Increasing evidence suggests that FOXO1 activity is significantly lost in EC.^20^ As inactivation of FOXO1 appears to be a crucial step in tumorigenesis, restoring or targeting FOXO1 to the cell nucleus represents a potential effective therapeutic strategy. Therefore, whether metformin, as an AMPK activator, regulates EC cell growth through the activation of FOXO1 needs further exploration.

Given the interrelationship between the AMPK and FOXO1 signaling pathways, we hypothesized that metformin may behave like a novel FOXO1 activator, with important chemotherapeutic implications for EC. The relevant AMPK–FOXO1 pathway proteins were assessed in endometrial samples. Furthermore, we examined the effect of metformin on proliferation and the AMPK–FOXO1 signal pathway using Ishikawa, HEC‐1B, and HHUA EC cell lines and a xenograft mouse model was established to study the significance of metformin in EC. We found that metformin suppressed EC proliferation through the regulation of the AMPK–FOXO1 signal pathway *in vitro* and *in vivo*.

## Materials and Methods

### Reagents

Metformin, compound C, and human recombinant insulin were from Sigma‐Aldrich (St. Louis, MO, USA). Anti‐pan‐FOXO1 (N18), anti‐ki‐67, and anti‐p‐AMPK (Thr172) antibodies were from Santa Cruz Biotechnology (Santa Cruz, CA, USA) for IHC. Anti‐p‐AMPK, anti‐pan‐AMPK (Thr172), anti‐p‐FOXO1 (Ser256), anti‐p‐AKT (Ser473), and anti‐pan‐AKT antibodies were from Cell Signaling Technology (Beverly, MA, USA). Anti‐pan‐FOXO1 (H128) antibody for Western blotting and immunofluorescence staining was from Santa Cruz Biotechnology. The BCA Protein Assay Kit for quantification of total protein and the Enhanced Chemiluminescence Detection Kit were from Pierce (Rockford, IL, USA). The Histostain‐Plus Kit was from Zhongshan Golden Bridge Biotechnology (Beijing, China). The annexin V–FITC/propidium iodide Apoptosis Detection Kit was from BD Biosciences (San Jose, CA, USA). The Lipofectamine 2000 reagent were from Invitrogen (Burlington, ON, Canada). The non‐specific scramble siRNA control and FOXO1‐specific siRNA were purchased from Cell Signaling Technology. All other chemicals were purchased from Sigma‐Aldrich.

### Tissue samples

We obtained 72 samples of formalin‐fixed and paraffin‐embedded tissue including 10 samples of NP tissue, 6 of SH, 5 of CH, 10 of CAH, and 41 of EC. The mean age of 41 patients with EC was 52 ± 1.6 years (range, 26–77 years). The EC samples were obtained from patients undergoing hysterectomy without preoperative chemotherapy or radiation and histologically validated for type and grade. The NP tissues were obtained by surgical excision or biopsy from patients with abnormal vaginal bleeding because of uterine fibroids, and the others were obtained from patients with abnormal endometrial biopsy confirmed by histological examination from a section of formalin fixed endometrial tissue.

We obtained 28 samples of EC (fresh tissue) by surgical excision and 9 NP samples (fresh tissue) from premenopausal women awaiting *in vitro* fertilization treatment because of tubal or male‐factor infertility or at the time of laparoscopy. The mean age of the 28 patients with EC was 53 ± 1.5 years (range, 36–72 years). The fresh tissues were frozen in liquid nitrogen immediately after resection and stored at −80°C until use. All tissue samples were obtained from the tissue bank of The First Affiliated Hospital of Shantou University Medical College (Shantou, China) after written consent from donors.

Use of these specimens was approved by the Ethics Committee of the Medical College, Guangdong Shantou University (Shantou, China). The project “The molecular mechanism of cell growth in EC regulated by AMPK–FOXO1 signal pathway” tracked the condition of EC patients and detected FOXO1, ki‐67 and p‐AMPK levels in tissue samples. The project conformed to the principles of the Chinese Ministry of Health application to measure human biomedical research and the Declaration of Helsinki. Endometrial samples were diagnosed double‐blinded by two pathologists (J.H. and L.H.) on the basis of the WHO classification. For consent for the tissue, the donors were informed that they voluntarily donated their tissues after the pathological diagnosis and the contract could be terminated at any time of they wished. Patients with newly diagnosed and pathologically confirmed EC were consecutively recruited from the First Affiliated Hospital of Medical College of Shantou University. Patients with a prior or concurrent malignancy were not included in the original study. Non‐human primates were not used in our research.

### Nude mice xenograft experiments

Six‐week‐old female BALB/c nu/nu mice weighing 20 ± 2 g were purchased from Vital River Laboratories (Beijing, China) and housed in the Laboratory Animal Center of Shantou University Medical College. Principles of standard laboratory animal care were followed, and all procedures were approved by the Animal Care Committee of our institute. The animals were randomized into control and experimental groups. HEC‐1B cells were resuspended in HBSS (Sigma) and s.c. injected (2 × 10^6^ cells/mouse) into each flank of 10 mice pretreated with an intragastric administration of metformin (200 mg/kg body weight; 0.1 mL/mouse) or isotonic saline for 2 weeks. Each group of mice was divided into two subgroups of five mice each. Tumors seen after 2 weeks were monitored every 3 days for growth and were collected 3 weeks after metformin treatment or an equivalent volume of isotonic saline. For intragastric administration, metformin was dissolved in physiological saline and given once daily at 200 mg/kg. The control group received isovolumic vehicle only. Tumor volume (*V*) was calculated as *V* = length × width^2^ × 0.52. All tumors were dissected from peritoneal surfaces and weighed. A representative portion of tumor was fixed in formalin, and the remainder was flash frozen and stored at −80°C.

### Cell culture

Ishikawa, HEC‐1B, and HHUA EC cell lines, were used in these experiments. The Ishikawa, HEC‐1B, and HHUA human cell lines were purchased from European Collection of Cell Cultures (cat. no. 99040201), ATCC (Rockville, MD, USA), and Riken Cell Bank (Tsukuba, Japan), respectively. All the three EC cell lines were grown in RPMI‐1640 medium supplemented with 10% FBS, 300 mM l‐glutamine, 10 000 U/mL penicillin, and 10 000 μg/mL streptomycin under 5% CO_2_.

### Cell growth

Ishikawa, HEC‐1B, and HHUA cells were trypsinized, counted, then plated in 24‐well plates at 1 × 10^4^ cells/well and allowed to incubate overnight. Cells were then treated with doses of metformin for 24–72 h. In experiments with AMPK inhibitor or insulin treatment, Ishikawa cells were cotreated with 2 mM metformin and compound C (15 μM) or insulin (10 nM) for 24–72 h. Cell growth was evaluated by counting the number of cells over time. Each experiment was undertaken in triplicate and repeated three times to assess consistency of results.

### Immunohistochemistry

Paraffin‐embedded, formalin‐fixed tissue was immunostained for FOXO1 or p‐AMPK or ki‐67 content with primary antibodies against FOXO1 (1:50 dilution) or p‐AMPK (1:100 dilution) or ki‐67 (1:100 dilution). After specimens were deparaffinized in xylene and graded alcohol, epitope retrieval was carried out: sections were heated in a microwave oven at 700 W for 20 min in 1× Antigen Retrieval Solution (Biogenex, San Francisco, USA). Then, endogenous peroxidase was blocked by immersing sections in 0.3% H_2_O_2_ methanol for 15 min. The reaction was visualized by use of the EnVision Detection Kit (Zhongshan Golden Bridge Biotechnology) with diaminobenzidine tetrahydrochloride as the enzyme substrate. All sections were counterstained with GM hematoxylin staining solution (Zhongshan Golden Bridge Biotechnology). All slides were reviewed independently by two investigators (J.H. and L.H.). Every tumor was given a score reflecting the mean intensity of the staining (0, no staining; 1, low staining; 2, medium staining; and 3, strong staining).^21^

### Annexin V analysis

Cells were cultured in 12‐well plates at 4 × 10^4^ cells/well for 24 h, then serum‐starved for 24 h and treated with various doses of metformin. Cells were trypsinized and resuspended in annexin‐binding buffer (10 mM HEPES [Invitrogen], 140 mM NaCl, and 2.5 mM CaCl_2_ [pH 7.4]) to approximately 1 × 10^6^ cells/mL. Annexin V, Alexa Fluor 647 conjugate (BD Biosciences), and DAPI (BD Biosciences) were added to each cell solution, and samples were analyzed by flow cytometry (Epics‐XLII; BD Biosciences) for early and late apoptosis.

### Western blot analysis

Ishikawa, HEC‐1B, and HHUA cells were trypsinized, counted, then plated in 6‐well plates at 2 × 10^5^ cells/well and allowed to incubate overnight. All of the three cell lines were deprived of serum for 18 h, then exposed to indicated doses of metformin for 24 h. In experiments with AMPK inhibitor, Ishikawa cells were pretreated with compound C (15 μM) for 5 h, and treated with 2 mM metformin for 24 h. In addition, Ishikawa cells were deprived of serum for 18 h, then exposed to metformin (2 mM) for 24 h and treated with regular human insulin (10 nM) for 15 min.

Human endometrium tissue (30 μg) and cells (5 × 10^5^) were sonicated in 300 μL RIPA buffer supplemented with protease and phosphatase inhibitor (3 μL), NaF (1 mM 3 μL), sodium orthovanadate (1 mM 3 μL), and PMSF (1 mM 3 μL), homogenized, then centrifuged (12 000 *g* for 5 min). Supernatant fractions (whole cell fractions) were collected and protein content was determined with the BCA Protein Assay Kit (Pierce). Proteins were separated by 10% SDS‐PAGE and transferred to nitrocellulose membranes, which were blocked in 5% non‐fat milk with TBS‐T at room temperature and incubated with primary antibodies for FOXO1, p‐FOXO1 (Ser 256), AMPK, p‐AMPK (Thr 172), Akt, p‐Akt (Ser 473) (Cell Signaling Technology, Danvers, MA, USA) in 1% nonfat milk overnight at 4°C. Membranes were washed three times with TBS‐T and incubated with a HRP‐conjugated secondary antibody at room temperature for 1 h, then washed and developed with the ECL Plus Western Blot Detection System kit (Amersham, Piscataway, NJ, USA) or the Supersignal West Dura Extended Duration Substrate (Pierce). The membrane was stripped and reprobed with antibody against GAPDH (1:10 000; Sigma‐Aldrich), pan‐FOXO1, pan‐AMPK, or pan‐Akt to confirm equal loading.

### Immunofluorescence staining

Cells were grown on glass coverslips and treated with 2 mM metformin for 24 h, then fixed with 4% paraformaldehyde, washed with phosphate‐buffered NaCl solution, and permeabilized with 0.1% Triton‐0.1% deoxycholate. Cells were blocked with 5% BSA in PBS. FOXO1 primary antibody (1:50 dilution; Santa Cruz Biotechnology) in 5% BSA was added to each sample and incubated overnight at 4°C in a humidified chamber, then with fluorescent anti‐rabbit IgG conjugates (1:50 dilution) for 1 h at room temperature in the dark. Cells were stained with DAPI for 3 min. Representative images were captured by a fluorescent microscope (Olympus BX‐50; Olympus, Japan).

### Quantitative real‐time PCR

The mRNA expression of FOXO1 was analyzed by RT‐PCR. The primer pairs were 5′‐TTATGACCGAACAGGATGATCT‐TG‐3′ (forward) and 5′‐TGTTGGTGATGAGAGAAGGTTGAG‐3′ (reverse). The efficiency of cDNA synthesis from each sample was estimated by RT‐PCR with β‐actin‐specific primers 5′‐GGCCAGGTCATCACCATTG‐3′ (forward) and 5′‐ATGATCTTGAGGCTGTTGTCATA‐3′ (reverse).

Total RNA was isolated from cells by use of RNAeasy (Qiagen) according to the manufacturer's protocol, and cDNA was synthesized from 500 ng RNA with use of the Omniscript RT kit (Qiagen) with random primers. Typically, 1 μL liquors of the reverse‐transcribed cDNA were amplified by 35 (FOXO1) or 35 (β‐actin) cycles of PCR. Each cycle consisted of denaturation at 94°C for 30 s, annealing at 52°C for 30 s, and extension at 72°C for 45 s for FOXO1 and denaturation at 95°C for 30 s, annealing at 52°C for 60 s, and extension at 72°C for 50 s for β‐actin.

### Small interfering RNA

Ishikawa cells were seeded overnight in the growth media in 6‐well plates and transfected the next day with 10 nmol/L non‐specific scramble siRNA control (#6568; Cell Signaling Technology) or FOXO1‐specific siRNA (#6256; Cell Signaling Technology) by use of Lipofectamine 2000 reagent (Invitrogen) according to the manufacturer's recommendations. At 48 h after transfection, cells were incubated in RPMI‐1640 medium supplemented with 10% FBS in the absence or presence of 2 mM metformin for 24 h. Cell number was counted before cells harvested with RIPA buffer for Western blot analysis of total protein. Silencing of FOXO1 was verified by Western blot analysis with FOXO1 antibody (1:1000; Cell Signaling Technology).

### Statistical analysis

Data are presented as mean ± SEM as appropriate. Statistical significance between two groups was determined by two‐tailed *t*‐test. Differences between tissue types were tested by one‐way anova followed by Dunnett's two‐tailed *t*‐test. *P* < 0.05 was considered statistically significant. All analyses involved use of spss 11.0 (SPSS, Chicago, IL, USA) or Prism 5.0 (GraphPad Software, San Diego, CA, USA).

## Results

### Phosphorylated AMPK and FOXO1 protein levels in normal and malignant endometrium

**Figure 1:**
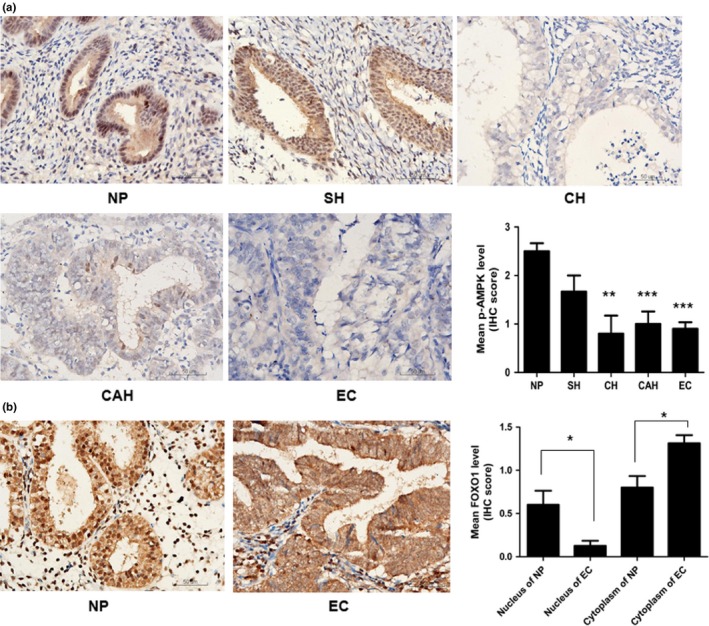
Downregulation of phosphorylated AMP‐activated protein kinase (p‐AMPK) and the nucleus human Forkhead box O1 (FOXO1) protein in malignant endometrium. (a) Immunohistochemical analysis of phosphorylated (p‐)AMPK protein in endometrial specimens: 10 normal proliferative (NP), 6 simple hyperplasia (SH), 5 complex hyperplasia (CH), 10 complex atypical hyperplasia (CAH), and 41 estrogen‐dependent endometrial cancer (EC) tissues. Strong staining of p‐AMPK in NP tissue but undetectable or markedly reduced in hyperplasia and particularly EC tissue. Histogram summarizes the outcome of statistical analysis of the staining results. (b) Protein immunoreactions of FOXO1 in endometrial specimens: 10 NP and 32 EC tissue. FOXO1 protein was observed in EC tissue within the cytoplasm with weak staining in the nucleus compared with NP tissue. Histogram summarizes the outcome of statistical analysis of the staining results. *P < 0.05; **P < 0.01; ***P < 0.001. Magnification, ×400; bar = 50 μm.

To investigate the intracellular signaling in EC, we determined the level of p‐AMPK in endometrial tissues (NP, *n* = 10; SH, *n* = 6; CH, *n* = 5; CAH, *n* = 10; and EC, *n* = 41) and FOXO1 protein localization in 32 cases of EC and 10 NP tissue samples by IHC. Phosphorylated AMPK staining was strong in NP (50%, 5/10) but was undetectable or markedly reduced in hyperplasia and particularly EC tissue (3%, 1/41) (Fig. 1a), which suggests that p‐AMPK levels decreased gradually with development of EC. FOXO1 protein was expressed in EC tissue within the cytoplasm with weak staining in the nucleus compared to normal controls (Fig. 1b), so FOXO1 protein in the nucleus was significantly reduced or absent in EC.

**Figure 2:**
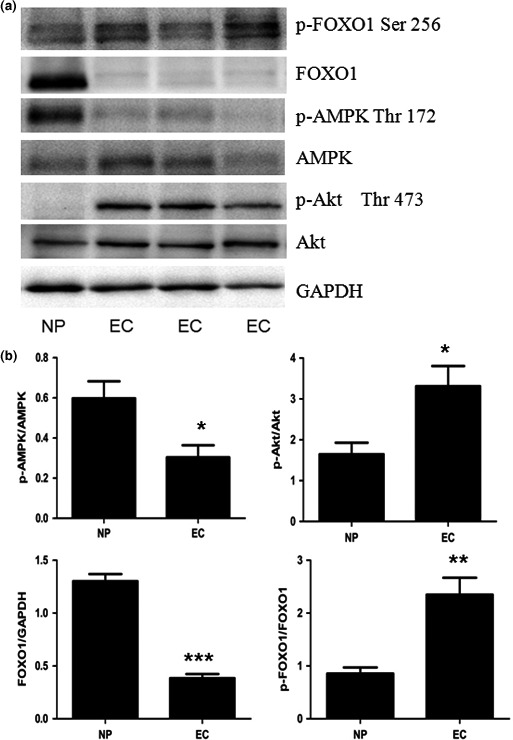
Western blot analysis of whole‐cell lysates of AMP‐activated protein kinase (AMPK), human Forkhead box O1 (FOXO1), or protein kinase B (Akt) expression in 9 normal endometrial (NP) samples and 28 estrogen‐dependent endometrial cancer (EC) tissue. GAPDH staining was a loading control. Phosphorylated (p‐)AMPK and FOXO1 protein levels were lower in EC than NP tissue, with no significance on p‐FOXO1; however, levels of p‐Akt, which facilitates nuclear export of FOXO1, were increased in EC tissues (a). Histogram summarizes the outcome of statistical analysis of the Western blotting results (b). *P < 0.05; **P < 0.01; ***P < 0.001.

To confirm our IHC results, we carried out Western blot analysis of 28 samples of EC and 9 of NP. Phosphorylated AMPK and FOXO1 protein levels were lower in EC than NP tissue, whereas the p‐FOXO1 level was increased in EC tissues (*P* < 0.05; Fig. 2), similar to those observed by IHC. Additionally, p‐Akt levels were increased in EC tissues (Fig. 2). Therefore, analysis of human samples indicated that a marked loss of AMPK and FOXO1 activity may play a central role in endometrial carcinogenesis.

### Metformin inhibits EC cell growth by activating AMPK

**Figure 3:**
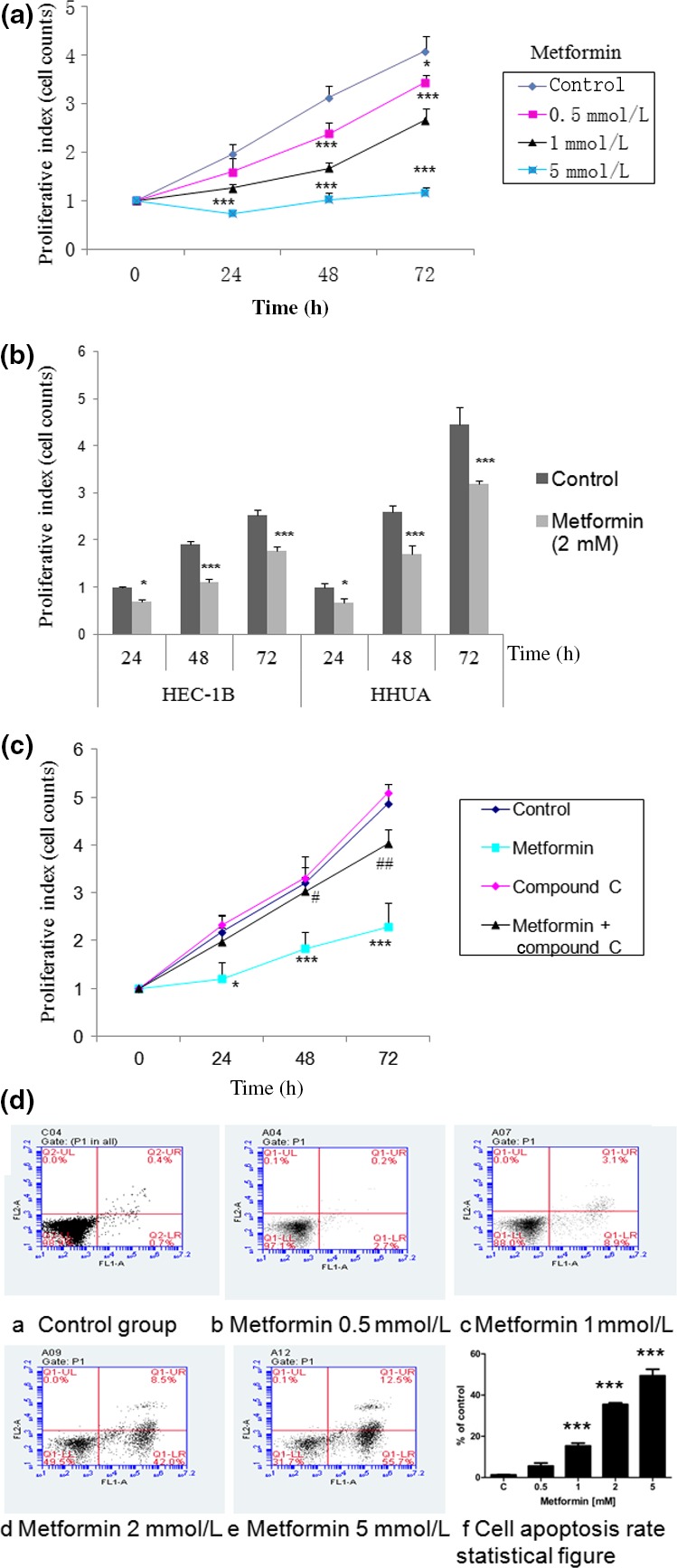
Effect of metformin on estrogen‐dependent endometrial cancer (EC) cell growth and apoptosis *in vitro*. Cells were grown in growth media with or without doses of metformin or 15 μM compound C for various times; cell numbers were counted to evaluate proliferative activity. Metformin significantly suppressed the proliferation of Ishikawa (a), HEC‐1B and HHUA cells (b), and compound C‐cancelled Ishikawa cell growth inhibition induced by metformin (c). (d) Effect of metformin on apoptosis. Ishikawa cells were treated with metformin for 24 h, and cell apoptosis was analyzed by annexin V staining. Treatment with metformin induced apoptosis in Ishikawa cells. Each point represents the means ± SEM of triplicate determinations in three independent experiments. *P < 0.05; **P < 0.01; ***P < 0.001, metformin‐treated versus control cells. #*P* < 0.05; ##*P* < 0.01, metformin + compound C‐treated versus metformin‐treated cells.

Ishikawa, HEC‐1B, and HHUA cell lines are widely used for the study of EC and were chosen for our study (Fig. 3). Metformin time‐dependently inhibited the growth of EC cells (Fig. 3a,b). To evaluate the mechanism of growth inhibition by metformin, Ishikawa cell growth was analyzed after treatment with the AMPK inhibitor, compound C. Compared to the untreated groups, the compound C (15 μM) treatment protected Ishikawa cells from metformin‐induced growth inhibition (Fig. 3c), suggesting that AMPK is directly involved in this process. Moreover, metformin induced apoptosis in Ishikawa cells (Fig. 3d).

### Metformin upregulated FOXO1 activity by AMPK activation in EC cells

**Figure 4:**
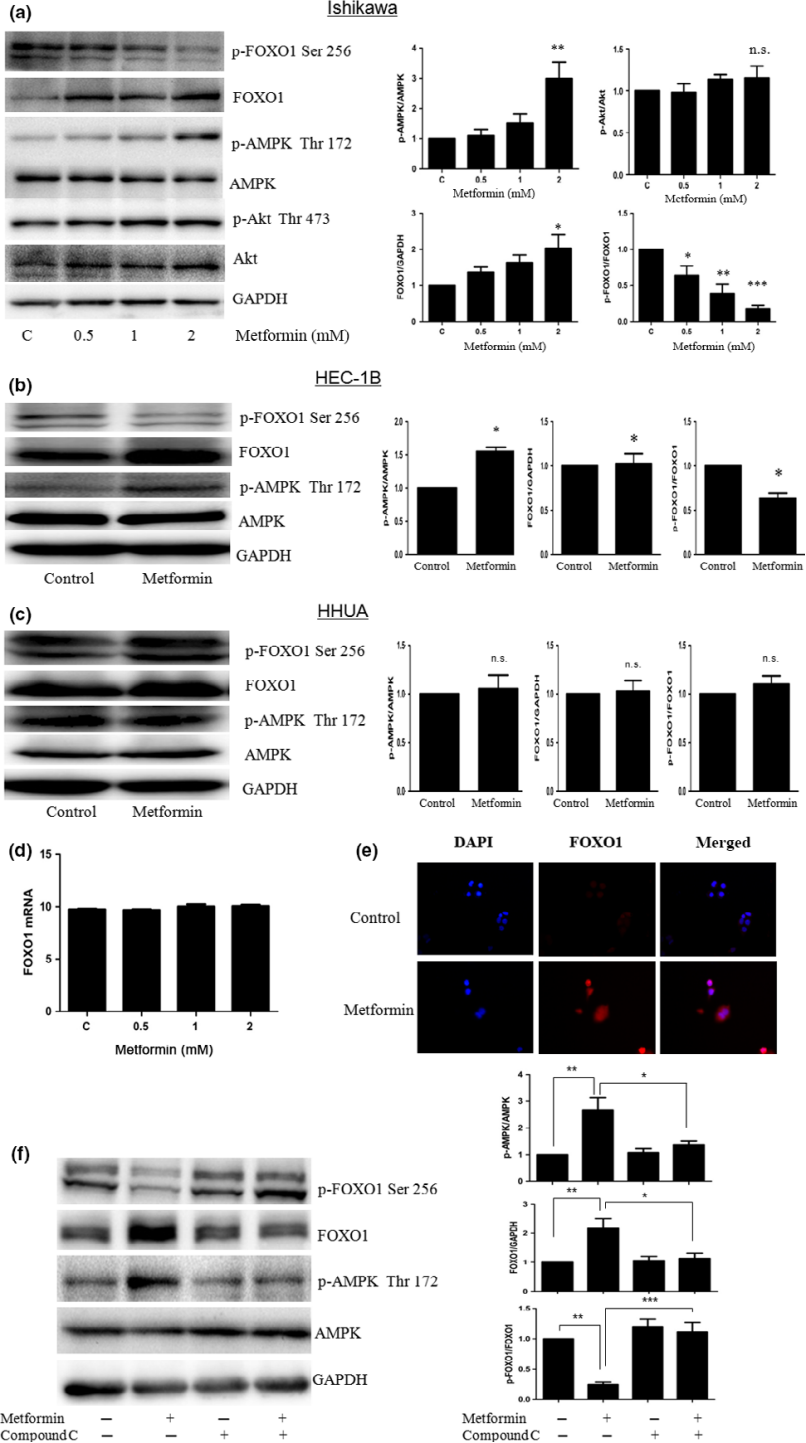
Metformin increased human Forkhead box O1 (FOXO1) activity by AMP‐activated protein kinase (AMPK) activation in endometrial cancer cells. Cells were treated with different doses of metformin for 24 h, then examined by Western blot for AMPK, FOXO1, and protein kinase B (Akt) protein level, or RT‐PCR for FOXO1 mRNA level. Metformin caused AMPK activation, decreased FOXO1 phosphorylation, and increased total FOXO1 protein in Ishikawa and HEC‐1B cells (a,b), but metformin had no effect on AMPK or FOXO1 protein levels in HHUA cells (c). Phosphorylated (p‐)Akt levels were not reduced by metformin in Ishikawa cells (a). (d) Quantitative real‐time PCR analysis indicated only minimally induced FOXO1 mRNA levels after treatment with metformin. (e) Immunofluorescence staining of FOXO1 protein localization in Ishikawa cells. FOXO1 staining (red) in the nucleus was further increased by 2 mM metformin. Magnification, ×400. (f) Ishikawa cells were pretreated with 15 μM compound C for 5 h, then incubated with 2 mM metformin for 24 h. Western blot showed, with p‐AMPK blocking, p‐FOXO1 increased and FOXO1 decreased by AMPK inhibitor, compound C. *P < 0.05; **P < 0.01; ***P < 0.001. n.s., no significance.

To examine whether metformin, an AMPK activator, regulates the proliferation of EC cells by forced nuclear accumulation of FOXO1, we characterized the effect of metformin on AMPK and FOXO1 activity. Metformin caused AMPK activation, decreased p‐FOXO1, and increased FOXO1 protein in Ishikawa and HEC‐1B cells (Fig. 4a,b), but not in HHUA cells (Fig. 4c), within 24 h of exposure.

We then investigated the molecular mechanism of FOXO1 induction with metformin in Ishikawa cells. FOXO1 mRNA expression was only minimally induced by metformin (Fig. 4d). Thus, metformin directly activated *de novo* protein synthesis without increasing FOXO1 mRNA expression. So, the localization of FOXO1 protein in Ishikawa cells was tested after metformin treatment (2 mM) by immunofluorescence staining. Findings confirmed that intensified staining of FOXO1 (red) in the nucleus in response to the metformin treatment (Fig. 4e). We next examined the level of p‐Akt, which regulates the subcellular localization of FOXO1. Levels of p‐Akt, which facilitates nuclear export of FOXO1, were not reduced by metformin (Fig. 4a). Therefore, metformin enhanced FOXO1 activity through post‐transcriptional modifications of FOXO1, but did not decrease Akt phosphorylation.

Hence, to determine whether AMPK pathways are involved in the metformin‐induced upregulation of FOXO1 activity, compound C was used to selectively inhibit AMPK activation. Ishikawa cells were pretreated with compound C and then incubated with metformin (2 mM) for 24 h. Findings confirmed that the metformin‐induced changes of FOXO1 and FOXO1 phosphorylation were partially cancelled by compound C in Ishikawa cells (Fig. 4f). These results suggest that metformin enhanced FOXO1 activity, at least in part, by activating AMPK.

**Figure 5:**
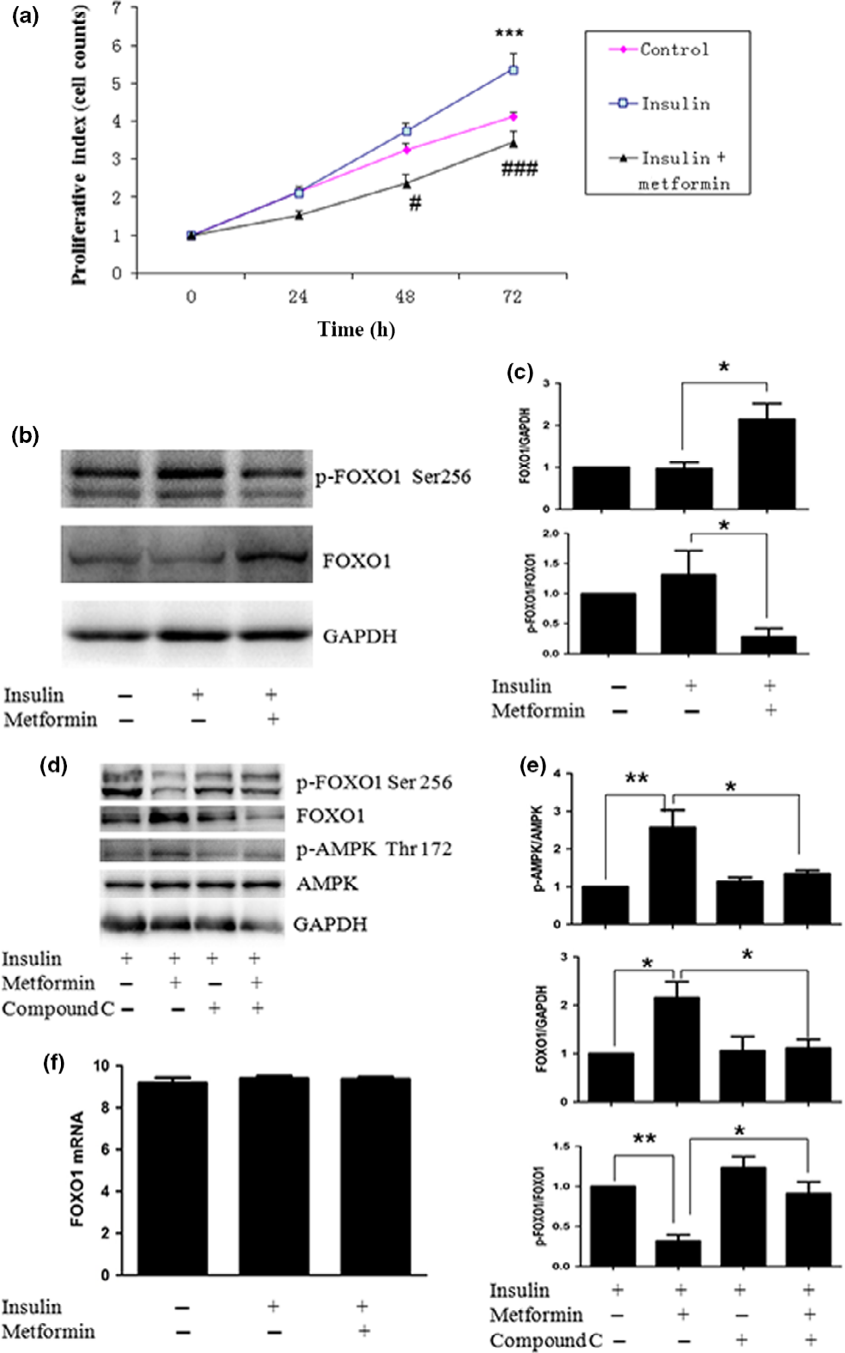
Effect of metformin on cell growth and the regulatory proteins in Ishikawa endometrial cancer cells with or without insulin. Cells were grown in growth media with or without 2 mM metformin or 10 nM insulin for various times; cell number was counted to evaluate proliferative activity. Metformin significantly inhibited cell growth induced by insulin (a). Ishikawa cells were treated with metformin for 24 h, then stimulated with insulin for 15 min and cells were examined by Western blot for human Forkhead box O1 (FOXO1) and phosphorylated (p‐)FOXO1, or RT‐PCR for FOXO1 mRNA. Metformin decreased FOXO1 phosphorylation induced by insulin represented by Western blot (b,c). (d,e) Cells were pretreated with 15 μM compound C for 5 h, treated with metformin for 24 h, then stimulated with insulin for 15 min. Cells were analyzed by Western blot for FOXO1 and AMP‐activated protein kinase (AMPK). Western blot analysis indicated that, with p‐AMPK protein blocking, p‐FOXO1 protein was increased and FOXO1 protein level was decreased after treatment with compound C. (f) Quantitative real‐time PCR analysis showed only minimally induced FOXO1 mRNA levels (without significance) after treatment with metformin or insulin. *P < 0.05; **P < 0.01; ***P < 0.001, insulin‐treated versus control cells. #*P* < 0.05, ###*P* < 0.001, insulin + metformin‐treated versus insulin‐treated cells.

Finally, we examined the effect of metformin on insulin‐induced proliferation in Ishikawa cells, as insulin was reported to be mitogenic and anti‐apoptotic in various *in vitro* cancer models, including EC cell lines. Metformin potently inhibited the growth of Ishikawa cells induced by insulin (Fig. 5a) and metformin increased the FOXO1 protein level by activating AMPK in the presence of 10 nM insulin (Fig. 5b–e) with no significant effect on FOXO1 mRNA (Fig. 5f), which is similar to findings with metformin alone. Taken together, these results suggest that metformin modulates FOXO1 activity through AMPK activation with or without insulin.

### Human Forkhead box O mediated metformin to inhibit EC cell growth

**Figure 6:**
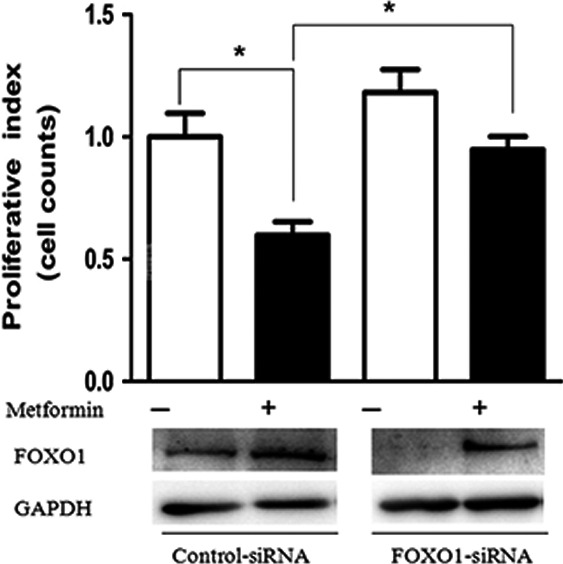
Role of human Forkhead box O1 (FOXO1) in the action of metformin. Estrogen‐dependent endometrial cancer (EC) cells were transfected with non‐specific scramble siRNA control or FOXO1‐specific siRNA. At 48 h after transfection, cells were incubated with or without metformin at 2 mM for 24 h. Then Western blot analysis was carried out to confirm FOXO1 protein levels. Simultaneously, cell numbers were counted in paired samples and showed a relative value (proliferation index) in each sample to evaluate the effect of metformin. *P < 0.05.

Our data showed that metformin suppressed EC cell growth and increased total FOXO1 protein. To determine whether FOXO1 contributes to metformin‐inhibited growth, we knocked down FOXO1 in Ishikawa cells by using siRNA. Cells were transfected with siRNA against FOXO1 and treated with or without metformin at 2 mM, then cell growth was monitored. Western blot analysis confirmed that the knockdown was successful, showing decreased FOXO1 expression. These cells showed significantly induced FOXO1 expression following treatment with metformin. In the absence of metformin, cells with FOXO1 siRNA knockdown showed increased growth compared with control siRNA treatment, so endogenous FOXO1 plays some role in cell proliferation. Metformin treatment significantly inhibited the growth of cells transfected with control siRNA, but the inhibited growth was largely abrogated in those with FOXO1 siRNA knockdown (Fig. 6). Thus, the effect of metformin was attenuated by FOXO1 knockdown, which supports the role of FOXO1 in metformin action. Knockdown of FOXO1 effectively cancelled metformin‐inhibited cell growth.

### Metformin inhibits the growth of EC in nude mice xenografts

**Figure 7:**
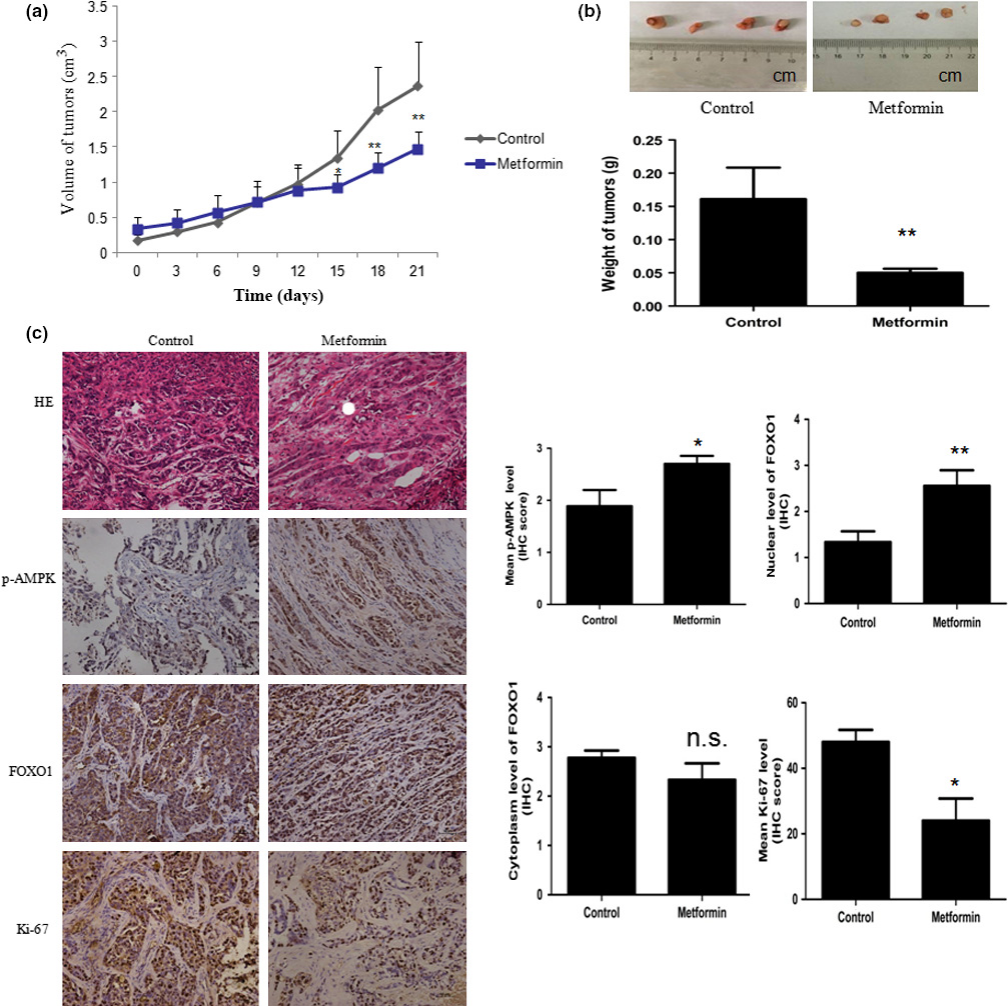
Metformin inhibited the growth of HEC‐1B endometrial carcinoma cell xenografts in nude mice. Xenografts were generated by the s.c. implantation of HEC‐1B cells, and mice were pretreated with intragastric metformin or physiological saline for 2 weeks. Two weeks after implantation, physiological saline (control) or metformin (200 mg/kg) were given intragastrically and the feeding continued until the end of the study. Tumor volumes and the weight of the animals were measured every 3 days. (a) Average tumor volumes of the HEC‐1B cell xenografts. (b) Photographs showing representative xenograft tumors on metformin‐ or vehicle‐treated nude mice (bar = 1 cm). Histograms show the average tumor volumes from the two groups. (c) Paraffin sections of excised tumors were assessed by H&E staining and immunohistochemistry showing the AMP‐activated protein kinase/human Forkhead box O1 (AMPK–FOXO1) pathway and proliferation index in both experiment and control groups. Magnification, ×200; bar = 50 μm. Phosphorylated (p‐)AMPK level and the nuclear level of FOXO1 were significantly increased in metformin‐treated HEC‐1B tumors compared to vehicle‐treated mice, with no significant decrease in the cytoplasm of FOXO1. Immunohistochemistry (IHC) showed a clear reduction in ki‐67 expression with a significant mean reduction in percentage of cells staining for ki‐67 in metformin‐treated tumors. Data represent mean ± SE. *n* = 10. *P < 0.05; **P < 0.01 versus control.

The *in vitro* results indicated that metformin induces the growth inhibition of EC cells by enhancing FOXO1 activity; we further examined the growth‐inhibitory effect of metformin in nude mice xenografts. Tumors developed in all 10 (100%) mice injected with HEC‐1B cells. Compared with no treatment, metformin suppressed HEC‐1B tumor growth and significantly decreased mean tumor weight (0.05 *vs* 0.16 g; *P* = 0.03) (Fig. 7a,b). Immunohistochemistry for p‐AMPK and FOXO1 was carried out to confirm modulation of the AMPK–FOXO1 pathway in tumor xenografts. Consistent with *in vitro* results, p‐AMPK levels and nuclear levels of FOXO1 (mean IHC score 1.3 *vs* 2.5; *P* = 0.009) were significantly increased in metformin‐treated HEC‐1B tumors compared to vehicle treatment, with no significant decrease in the cytoplasm of FOXO1 (Fig. 7c). In addition, ki‐67 protein expression was decreased, shown by IHC, with a significant mean reduction in the proportion of cells stained for ki‐67 in metformin‐treated HEC‐1B tumors (24%; *P* = 0.015) (Fig. 7c).

Because the dosage of metformin (200 mg/kg/day) used in the *in vivo* study was much higher than that used for diabetes treatment (850 mg/day), the weight of the animals was measured to evaluate possible side‐effects caused by metformin. The metformin treatment had no significant effect on the body weight of animals during treatment (data not shown).

## Discussion

The chemopreventive and antineoplastic effects of metformin are currently being evaluated for the treatment of several cancers. Although its mechanism of action is not fully understood, metformin is thought to inhibit cell proliferation locally through activation of the AMPK signaling pathway. In this study, we investigated the mechanism underlying the antitumorigenic effect of metformin in EC cells. Analysis of human samples showed a marked loss of AMPK and FOXO1 activity in EC compared to normal controls (Figs 1 and 2). Our findings showed that metformin suppressed the growth of three EC cell lines tested *in vitro* (Fig. 3). In addition, metformin decreased FOXO1 phosphorylation and increased FOXO1 protein levels in Ishikawa and HEC‐1B cells through AMPK activation (Fig. 4). Similar results were obtained after treatment with insulin (Fig. 5). Moreover, FOXO1 siRNA knockdown effectively cancelled metformin‐inhibited cell growth (Fig. 6). A xenograft mouse model provided further evidence of metformin in EC growth inhibition *in vivo* (Fig. 7). Thus, metformin may function as a novel FOXO1 activator for both the treatment and prevention of EC.

This study shows that AMPK and FOXO1 activity are indeed almost eliminated in EC, as indicated by IHC and Western blot analysis (Figs 1 and 2). Loss of Lkb1 protein in EC cells downregulated AMPK signaling downstream of Lkb1, the kinase responsible for phosphorylating and activating AMPK.^22^ From a clinical perspective, dysregulation of FOXO transcription factors has been described in several cancers, including EC, prostate, breast, and colon cancers.^20^, ^23^, ^24^, ^25^, ^26^ Additionally, the phosphorylation of FOXO1 by Akt leads to its inactivation by translocation from the nucleus to the cytoplasm.^27^, ^28^, ^29^, ^30^ In agreement with these reports, our results show that EC cells express high levels of Akt, thus FOXO1 is primarily distributed in the cytoplasm (Figs 1 and 2). However, in Western blot analysis, FOXO1 protein levels were significantly decreased in tumors (Fig. 2). These data were further supported by an earlier study showing almost undetectable FOXO1 immunoreactivity by tissue microarray of EC and a marked reduction in FOXO1 activity in EC, which is, at least in part, due to impaired FOXO1 mRNA expression.^31^ Therefore, we speculate that loss of AMPK and FOXO1 activity may play a crucial role in EC development.

A recent study reported that short‐term presurgical metformin reduces cellular proliferation in CAH and endometrioid EC.^32^ Use of metformin in obese women with EC is associated with a reduced incidence of cancer recurrence and EC patients receiving metformin were less likely to recur than those not on the drug.^33^ In agreement, our study indicated that metformin suppressed the growth of three tested EC cell lines (Fig. 3). Metformin accumulates in the tumor tissue and activates AMPK, which is a central energy sensor and regulator of cellular and whole‐body energy homeostasis.^14^

**Figure 8:**
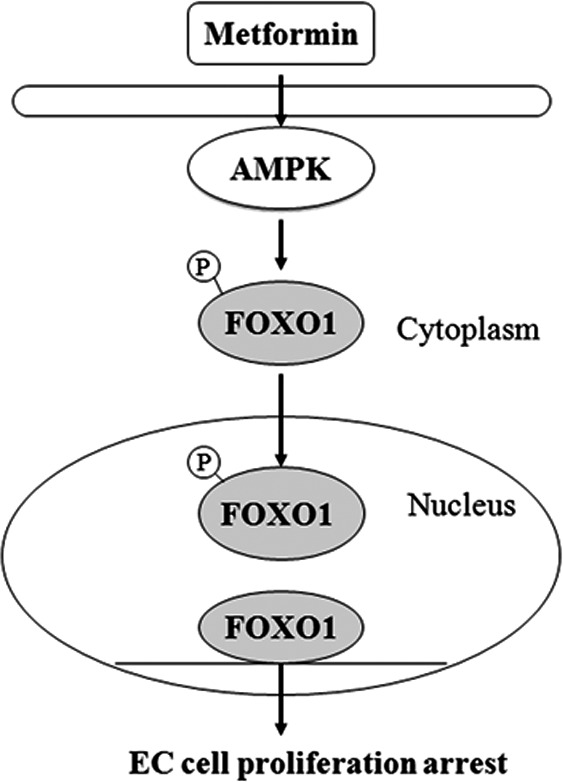
Proposed scheme for the antiproliferative mechanism of metformin in estrogen‐dependent endometrial cancer (EC) cells. Metformin inhibits EC cell proliferation by activating the AMP‐activated protein kinase/human Forkhead box O1 (AMPK–FOXO1) pathway. Metformin may exert its antiproliferative effect by activating AMPK and thus decreasing phosphorylation (p) of FOXO1 protein, thereby triggering the relocalization of FOXO1 protein from the cytoplasm to the nucleus and resulting in increased FOXO1 activity.

The AMPK–FOXO pathway plays a crucial role in the ability of a dietary restriction regimen to extend lifespan in *Caenorhabditis elegans*. Expression of constitutively active AMPK extended lifespan and resistance to oxidative stress, in a FOXO‐dependent manner.^34^ It has been reported that FOXO1 forms complexes with other proteins to indirectly regulate gene expression, but the nuclear localization of FOXO1 is a prerequisite for this gene modulation function.^35^, ^36^ The subcellular localization of FOXO1 is dependent on its post‐translational modifications, particularly its phosphorylation status.^37^, ^38^, ^39^, ^40^ In the phosphorylated state, FOXO1 is excluded from the nucleus and degraded in the cytoplasm in a ubiquitin‐dependent manner. We examined AMPK and FOXO1 activity, and found a significant decrease in p‐FOXO1 and a significant increase in FOXO1 proteins, but no significant change on FOXO1 mRNA expression, in response to metformin, which suggests that metformin enhances FOXO1 activity through the post‐transcriptional modification of FOXO1 (Fig. 4). These results are in harmony with an early report showing that metformin augments the FOXO1‐ and p53‐mediated endothelial cell senescence and apoptosis, through maintaining/restoring SIRT1 protein expression, which attenuated FOXO1 acetylation and increased FOXO1 activity.^41^ Our findings further confirmed that AMPK pathways are involved in the metformin‐induced upregulation of FOXO1 activity in Ishikawa cells, supported by a study indicating that AMPK activates FOXO/DAF‐16‐dependent transcription and phosphorylates FOXO/DAF‐16, suggesting a possible direct mechanism of regulation of FOXO/DAF‐16 by AMPK.^42^ It has previously been shown that phosphorylation of FOXO1 at Ser‐256 by Akt is not only responsible for its translocation from the nucleus to the cytoplasm but is also required for Skp2 (an oncogenic subunit of the Skp1/Cul1/F‐box protein ubiquitin complex) ubiquitination and degradation of FOXO1.^43^ In the present study, Akt and Akt phosphorylation were not affected in Ishikawa cells with metformin treatment (Fig. 4a), which was in agreement with a previous study.^44^ Collectively, these data suggest that activation of AMPK directly prevents the phosphorylation of FOXO1 but not decreased Akt phosphorylation, resulting in nuclear retention during the post‐translational modifications of FOXO1. Therefore, metformin may exert its antiproliferative effect by activating AMPK and thus decreasing phosphorylation of FOXO1 protein, thereby triggering the relocalization of FOXO1 protein from the cytoplasm to the nucleus and resulting in increased FOXO1 activity. Our model is summarized schematically in Figure 8.

The cancellation of the metformin‐induced upregulation of FOXO1 by compound C, a known inhibitor of the AMPK pathway, was only partial (Fig. 4f). Likewise, FOXO1 siRNA knockdown only partially cancelled the suppressive action of metformin (Fig. 6). These results suggest that metformin increases FOXO1 activity through both AMPK–FOXO1‐dependent and other independent pathways. Although in our HHUA cells, metformin suppressed cell growth, the changes in FOXO1 and FOXO1 phosphorylation were not caused by metformin, which suggests that metformin inhibited cell growth through other AMPK–FOXO1‐independent pathways. The cellular and molecular mechanisms responsible for the action of metformin are complicated and, in some cases, appear to extend beyond the AMPK activator. This observation explains, to some extent, why in some carcinoma cells metformin exerts its antitumor action through an AMPK‐independent pathway. Altogether, metformin increased FOXO1 activity in Ishikawa and HEC‐1B cells, which to our knowledge is the first time metformin has been linked to the regulation of FOXO1 activity.

The *in vivo* study further verified that metformin suppresses tumor growth, accompanied by downregulated ki‐67 and upregulated AMPK phosphorylation and nuclear FOXO1 protein (Fig. 7). Other animal experiments have shown that metformin prevents the development of tumorigenesis in the pancreas, lung, and liver.^45^, ^46^, ^47^ However, the preventive use of metformin in the HEC‐1B EC xenograft model failed to prevent tumorigenesis, but significantly suppressed tumor development (Fig. 7a,b). Our previous study also found that the preventive use of metformin significantly delayed tumor development in an esophageal squamous cell carcinomas xenograft model.^48^

In the present study, AMPK and FOXO1 activity was eliminated in EC, and metformin exerted its antiproliferative effect by upregulating FOXO1 activity in Ishikawa and HEC‐1B cells. However, clinical studies are needed to validate the efficacy of metformin‐induced FOXO1 upregulation in ECs. The role of FOXO1 in tumorigenesis is further supported by findings in prostate cancer and cervical cancer.^49^, ^50^ As the role of FOXO1 protein in tumorigenesis becomes increasingly apparent, a study of direct FOXO1 activator use in EC should be considered and prospective clinical research is necessary to fully appreciate the impact of a direct FOXO1 activator on tumor regression and patient survival.

Taken together, the present data imply that FOXO1 may be upregulated through metformin‐induced AMPK (Fig. 8). The present results also imply that enhancing FOXO1 function contributes to increased efficacy of metformin therapy against EC. These studies reveal a novel mechanism of antineoplastic effect for metformin, and suggest that the AMPK–FOXO1 pathway may be a therapeutic target for the development of new antineoplastic drugs.

## Disclosure Statement

The authors have nothing to disclose.
